# Klotho Exerts an Emerging Role in Cytokinesis

**DOI:** 10.3390/genes11091048

**Published:** 2020-09-04

**Authors:** Chiao-Yin Sun, Chi-Yuan Chou, Yu-Ying Hsieh, Kang-Chieh Lo, Yan-Liang Liou, Yau-Hung Chen

**Affiliations:** 1 Department of Nephrology, Keelung Chang Gung Memorial Hospital, Keelung 204, Taiwan; fish3970@gmail.com (C.-Y.S.); yutinghsieh0913@gmail.com (Y.-Y.H.); 2 College of Medicine, Chang Gung University, Taoyuan 333, Taiwan; 3 Department of Life Sciences and Institute of Genome Sciences, National Yang-Ming University, Taipei 112, Taiwan; 4 Medical Research Center, Keelung Chang Gung Memorial Hospital, Keelung 204, Taiwan; 5 Department of Chemistry, Tamkang University, Tamsui, New Taipei City 251, Taiwan; bugisme780930@gmail.com (K.-C.L.); a0989833750@gmail.com (Y.-L.L.)

**Keywords:** Klotho, cell cycle, mitosis, cytokinesis, Aurora kinases, citron kinase

## Abstract

The Klotho gene functions as an anti-aging gene. A previous klotho-knockout mice study indicated that neither male nor female gametocytes could accomplish the first meiotic division. It suggested that Klotho might regulate cell division. In this study, we determined the roles of Klotho in cytokinesis in cultural human cells (HEK293 and HeLa) and in zebrafish embryos. Immunoprecipitation, mass spectrometry analysis, and a zebrafish model were used in this study. The results showed that Klotho is located in the midbody, which correlated with cytokinesis related kinases, Aurora kinase B and citron kinases, in the late stage of cytokinesis. There was a spatial correlation between the abscission site and the location of Klotho in the cytokinesis bridge. A three-dimensional structural reconstruction study demonstrated there was a spatial correlation among Klotho, Aurora kinase B, and citron kinases in the midbody. In addition, Klotho depletion inactivated Aurora kinases; it was also indicated that Klotho depletion caused aberrant cell cycle and delayed cytokinesis in a cell model. The study with zebrafish embryos suggested that klotho knockdown caused early embryo development abnormality due to dysregulated cytokinesis. In conclusion, Klotho might have a critical role in cytokinesis regulation by interacting with the cytokinesis related kinases.

## 1. Introduction

Klotho is a type-I membrane protein related to β-glucuronidases, which is encoded by the KL gene in humans [1]. Klotho is known as an anti-aging gene. Klotho-deficient mice manifest a syndrome resembling accelerated human aging, and display extensive and accelerated arteriosclerosis [2]. It has also been demonstrated that an overexpression of Klotho in mice might extend their average life span compared with normal mice [3]. It was observed that perturbing insulin and IGF1 signaling caused aging-like phenotypes in Klotho-deficient mice, and that the Klotho-mediated inhibition of insulin and IGF1 signaling contributed to its anti-aging properties [3]. Clinically, variations in Klotho function have been reported to contribute to heterogeneity in the onset and severity of human age-related phenotypes [4].

It is also known that Klotho has a critical role in the calcium–phosphate hemostasis. The bone-derived growth factor, FGF23, was associated with parathyroid hormone and vitamin D, and mobilizes the sodium–phosphate cotransporters that control renal phosphate transport in proximal tubular epithelial cells. In the presence of Klotho, FGF23 activated its receptor, which influences the homeostasis of phosphate. As a co-receptor of FGF, Klotho converted canonical FGFR1 into a specific receptor for FGF23 [5,6,7]. Furthermore, the beta-glucuronidase activity of Klotho could activate the cell surface calcium channel (TRPV5) by hydrolysis of its extracellular N-linked oligosaccharides [8].

In addition to aging and calcium–phosphate hemostasis, accumulated evidence suggests that Klotho is a multifunctional protein. Recent studies have indicated that Klotho might have critical roles in inflammation, oxidative stress, glucose metabolism, cell proliferation, and apoptosis [9,10,11,12,13]. However, the cytological role of Klotho is still a controversial issue. Therefore, the potential molecular mechanisms of Klotho were further investigated in the present study. 

Cytokinesis is the final stage of the cell division process during which the cytoplasm of a single eukaryotic cell divides into two daughter cells. Actin filaments and myosin-II motors, as well as many other proteins, are reported to function in the cytokinesis. Several kinases, such as Aurora kinases and polo kinase, have important regulatory roles in cytokinesis [14,15]. Previous studies have suggested that Klotho depletion might impair the division of gonadal cells and impair the maturation of gonadal cells [2]. Recent studies also indicate that Klotho has important roles in cell proliferation and differentiation [9]. It is hypothesized that Klotho might have roles in the cell division process. Our study results demonstrated that Klotho might have a critical role in cytokinesis regulation by interacting with the cytokinesis related kinases.

## 2. Materials and Methods 

### 2.1. Cell Culture and Transfection

The human embryonic kidney (HEK) 293 cell lines and Hela cells were purchased from American Type Culture Collection (Manassas, VA, USA). All of the cell lines were maintained in a DMEM medium (Sigma-Aldrich, St. Louis, MO, USA) containing 10% FBS (Sigma-Aldrich, St. Louis, MO, USA), and were supplemented with antibiotics and cultured in a humidified atmosphere (95% O_2_, 5% CO_2_) at 37 °C. For the cell cycle synchronization study, the cultured cells were treated with serum free medium or nocodazole (0.4 mg/mL; Sigma-Aldrich, St. Louis, MO, USA) for 16 h [16].

The plasmids Klotho-sh-green fluorescent protein (GFP; Cat. No: TL303678, OriGene, Rockville, MD, USA), membrane Klotho-V5, and secreted Klotho-V5 (Plasmid #17712, Plasmid #17713, Addgene, Cambridge, MA, USA) were used for the Klotho knock down and overexpression. HEK293 and HeLa cells (1 × 10^5^) were seeded in each well and cultured for 24 h at 37 °C. The cells were then co-incubated with 10 µL Lipofectamine^®^ 2000 (Thermo Fisher Scientific, Inc., Waltham, MA, USA) and 4 µg plasmids at 37 °C for 6 h. Subsequently, the cells were treated with fresh medium, followed by an additional 24 h of incubation at 37 °C. Following the transfection procedure, the cells with a GFP expression were selected with flow cytometry (FACSAriaIIu_2, Becton Dickinson, Franklin Lakes, NJ, USA) and cultured for the further studies.

### 2.2. Immunoprecipitation, Mass Spectrometry and Pathway Analysis

Each immunoprecipitation (IP) was performed in triplicate. HEK293 cells were grown on 10 cm plates until 90% confluent, and were then harvested. The harvested cells were lysed with 500 μL of 0.5% NP40 lysis buffer, 50 mM HEPES pH 7.6, 150 mM KCl, 2 mM EDTA, 0.5% NP40, and 0.5 mM DTT, and protease/phosphatase (Sigma-Aldrich, St. Louis, MO, USA) at 4 °C for 15 min. The lysates were cleared using IgG–Agarose (Sigma-Aldrich, St. Louis, MO, USA). Then, 5 mg of the total protein was used for IP with 50 μg of either pre-cross-linked Klotho antibody (N terminal specific, Sigma-Aldrich, St. Louis, MO, USA) or non-specific IgG (Sigma-Aldrich, St. Louis, MO, USA). The IP samples were then digested using 20 ng/mL of trypsin (Sigma-Aldrich, St. Louis, MO, USA; 37 °C, overnight). Finally, the peptides were extracted, concentrated to dryness under vacuum, and stored at −20 °C until LC/MS/MS analysis. The type of search of mass spectrometer (Thermo LTQ-Orbitrap, Thermo Fisher Scientific, Waltham, VA, USA) was MS/MS ion search. The database search parameters were as follows: Database, SwissProt 2013_03 (539,616 sequences and 191,569,459 residues); enzyme, Trypsin; fixed modification, carbamidomethyl (C); variable modifications, oxidation (M); taxonomy homo species, human; mass values, monoisotopic; protein mass, unrestricted; peptide mass tolerance: ±10 ppm; fragment mass tolerance, ±1 amu; max missed cleavages, 2; and instrument type, ESI-TRAP. Proteins with >two-folded changes (compared with the control group) and two peptides identified by mass spectrometer were considered significant. 

The pathway analysis was done with Reactome V62 [17]. All of the non-human identifiers were converted to their human equivalents. Intact interactors were used to increase the analysis background. Pathways with a *p* value less than 0.05 were considered significant.

### 2.3. Western Blotting Analysis

The total protein was extracted using a commercial kit according to the manufacturer’s instructions (Protein Extraction Kit, Millipore, Billerica, MA, USA). Thirty micrograms of protein from each sample was mixed with a sample loading buffer and loaded onto separate lanes on a 12% sodium dodecyl sulfate-polyacrylamide gel. The proteins were electro-transferred onto polyvinylidene fluoride membranes (0.2 μm: Immun-Blot, Bio-Rad, Hercules, CA, USA) and then immunoblotted with primary antibodies. The intensity of each band was quantified using NIH Image software (Bethesda, MD, USA), and the densitometric intensity corresponding to each band was normalized against the β-actin expression. The primary antibodies used for immunoblot were as follows: rabbit polyclonal β-actin antibody (abcam, ab8227, 1:1000), rabbit polyclonal Klotho antibody (Sigma-Aldrich, St. Louis, MO, USA, SAB3500604, 1: 500), rabbit monoclonal cyclinB1 antibody (abcam, ab32053, 1:1000), Aurora kinase A/-p antibody (abcam, ab13824, ab18318, 1:1000), Aurora kinase B/-p antibody (Thermo Fisher Scientific, Waltham, VA, USA, MA5-15321, PA5-38557, 1:1000), and mouse polyclonal citron kinase antibody (Abnova, H00011113-A01, 1:1000).

### 2.4. Immunofluorescence Staining, Confocal Microscopy, Live Cell Image, and 3D Structure Reconstruction Analysis

For immunofluorescence staining, cultured HeLa cells fixed by methanol were incubated with primary antibodies, followed by incubation with a fluorescent secondary antibody. The primary antibodies used for immunofluorescence staining were as follows: rabbit polyclonal Klotho antibody (Sigma-Aldrich, St. Louis, MO, USA, SAB3500604, 1: 100), mouse monoclonal Aurora kinase B antibody (Thermo Fisher Scientific, Waltham, VA, USA, MA5-15321, 1:100), and mouse polyclonal citron kinase antibody (Abnova, H00011113-A01, 1:100). The fluorescent staining for tubulin was done with a tubulin-RFP fusion protein construct (Thermo Fisher Scientific, Waltham, VA, USA, CellLight^®^ Tubulin-RFP, BacMam 2.0). For the nucleus staining, cells were incubated with 4′,6′-diamidino-2-phenylindole hydrochloride (DAPI) (abcam). The stained samples were observed under a confocal microscope (Leica Microsystems, Bannockburn Ill, Wetzlar, Germany). Imaris software (version 7.6.5.; Bitplane, Concord, MA, USA) was used to generate a 3D model from the stacks that were acquired every 1 μm. Living cell images were observed using a Nikon Eclipse Ti-E (Nikon Instruments Inc., Melville, NY, USA) with TokaiHit WSKM chamber and were controlled by NIS-element AR. The objective lens used was 100× CFI Plan Apochromat Lambda 100× oil objectives (N.A.1.45). Time-lapse images were acquired at 3 s intervals for 2 min by Photometrics CoolSNAP MYO CCD.

### 2.5. Zebrafish Embryo Staging and Morpholino Injection

Mature zebrafish (wild type, WT; AB strain) were maintained at 28 °C with a standard protocol [18]. The embryos were produced using standard procedures and were staged according to standard criteria (hours postfertilization, hpf) or by days postfertilization (dpf) [19,20]. Antisense morpholino oligonucleotide (MO), Klotho-MO (targeting the translation initiation site; 5′-GCAGAGGAATCCATGTCACTTTCAT-3′), and control MO (5′-CCTCTTACCTCAGTTACAATTTATA-3′, random sequence) were designed and obtained from Gene Tools, Philomath, OR. The MOs were dissolved in a 1 × Danieau solution containing 0.5% Phenol red, and 2.3 nL of MO solution of the indicated concentration was injected into the one-cell-stage of wild-type embryos. Sixty embryos for each group were injected, and 10–12 of them were selected for taking videos. All of the animal handling procedures were approved by the Use of Laboratory Animal Committee, Tamkang University, Tamsui, New Taipei City, Taiwan (No. 105001). All of the animal experiments were performed in accordance with the relevant guidelines and regulations.

### 2.6. Statistical Analyses

All of the data were expressed as mean ± standard error. One-way analysis of variance with Bonferroni corrections was performed for analyzing the data of the cell culture study. The data of the different animal groups were compared using the Wilcoxon–Mann–Whitney test. *p*-values of <0.05 were considered statistically significant.

## 3. Results

### 3.1. Reactome Pathway Analysis of Klotho

**Figure 1:**
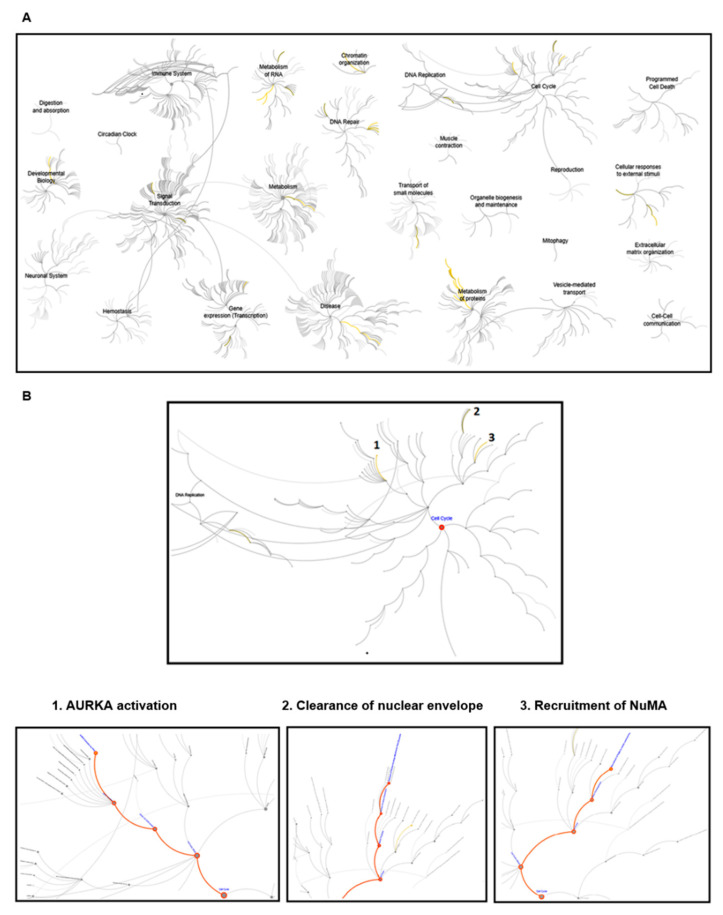
The results of the reactome pathway analysis. The protein extracts from the HEK293 cells were used for the immunoprecipitation study. There were 202 proteins with a two-fold increase (Klotho Ab vs. non-specific IgG) identified by the immunoprecipitation (IP)/LC/MS/MS analysis, and followed by reactome pathway analysis. (**A**) Plot of the pathways with *p* value less than 0.05. The significant pathways were marked as yellow lines. (**B**) Plots of the pathways of the cell cycle and mitosis. Three significant pathways of cell cycle and mitosis, Aurora kinase A (AURKA) activation, clearance of nuclear envelope, and recruitment of NuMA are shown and marked with orange lines.

For exploring the potential function of Klotho, an IP/LC/MS/MS study was performed (using HEK293 cell lysates). There were 202 proteins identified by the above analysis (Supplementary information, Table S1a). The reactome pathway analysis results are listed in the Supplementary Information (Supplementary information, Table S1b). These results suggested that Klotho had potential roles in multiple biological processes and diseases (Figure 1A). Several pathways, Aurora kinase A (AURKA) activation, clearance of nuclear envelope, and recruitment of NuMA, which are involved in cell cycle regulation and mitosis, were highlighted (Figure 1B). It was suggested that Klotho had potential roles in the cell cycle regulation and mitotic processes.

### 3.2. Klotho Depletion Caused Aberrant Cell Cycle 

**Figure 2:**
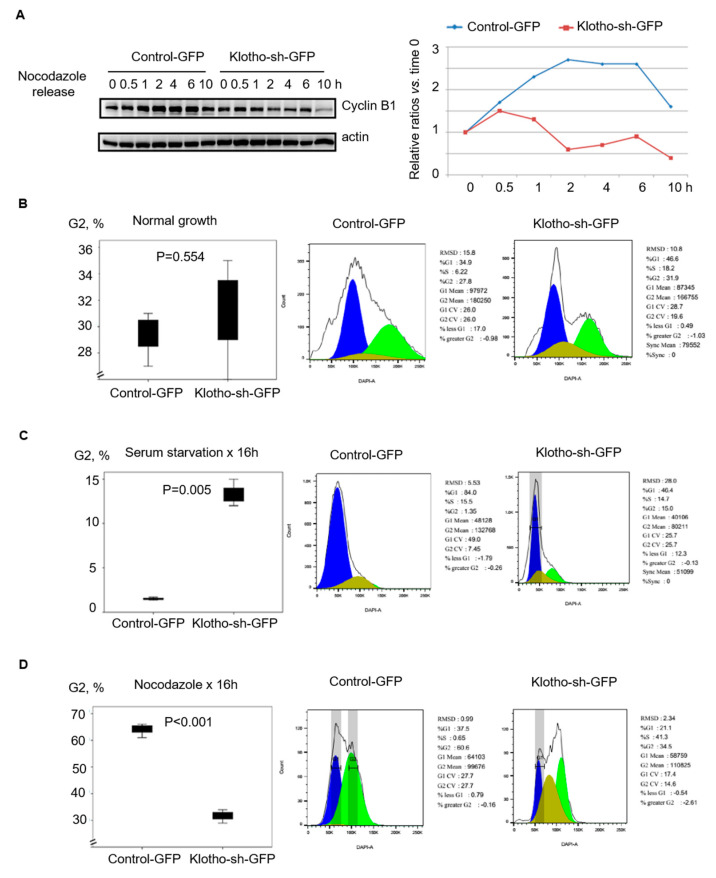
Klotho depletion causes aberrant cell cycle in HEK293 cells. HEK293 cells transfected with control-green fluorescent protein (GFP) or Klotho-sh-GFP for 24 h selected by flow cytometry were cultured with indicated condition, as indicated by the figure legends. (**A**) The protein expression of cyclin B1 was evaluated by Western blotting after treatment with nocodazole. (**B**–**D**) Cell cycle analysis after treatment with varying conditions, as indicated in the figure legends, was done with Propidium iodide staining followed by flow cytometry analysis. Each reaction was repeated in triplicate. Representative histograms of cell cycle distribution in all treatments are demonstrated (means ± standard error of the mean (SEM); one-way analysis of variance (ANOVA)).

For determining the roles of Klotho in the cell cycle and mitosis, cell cycle markers, cyclin B1 and cyclin D1, were evaluated by Western blotting with nocodazole-arrested mitotic HEK293 cell extracts. In these cells, we found that the G2/M marker, cyclin B1, increased after the cells were released from nocodazole and declined after 6 h in the cells transfected with the control-GFP. In contrast, the cyclin B1 levels started to decrease soon after nocodazole release (0.5 h) in the cells with Klotho depletion (Figure 2A). Following Klotho depletion by transfection with Klotho-sh-GFP, cell cycle distribution was determined by flow cytometry analysis. Under the normal culture conditions, the Klotho depleted cells had a similar cell cycle distribution as the control cells (Figure 2B,C). However, the cell cycle distribution of the Klotho depleted cells was significantly different from the control cells under synchronization with nocodazole (Figure 2D). These results indicated Klotho depletion caused an aberrant cell cycle.

**Figure 3:**
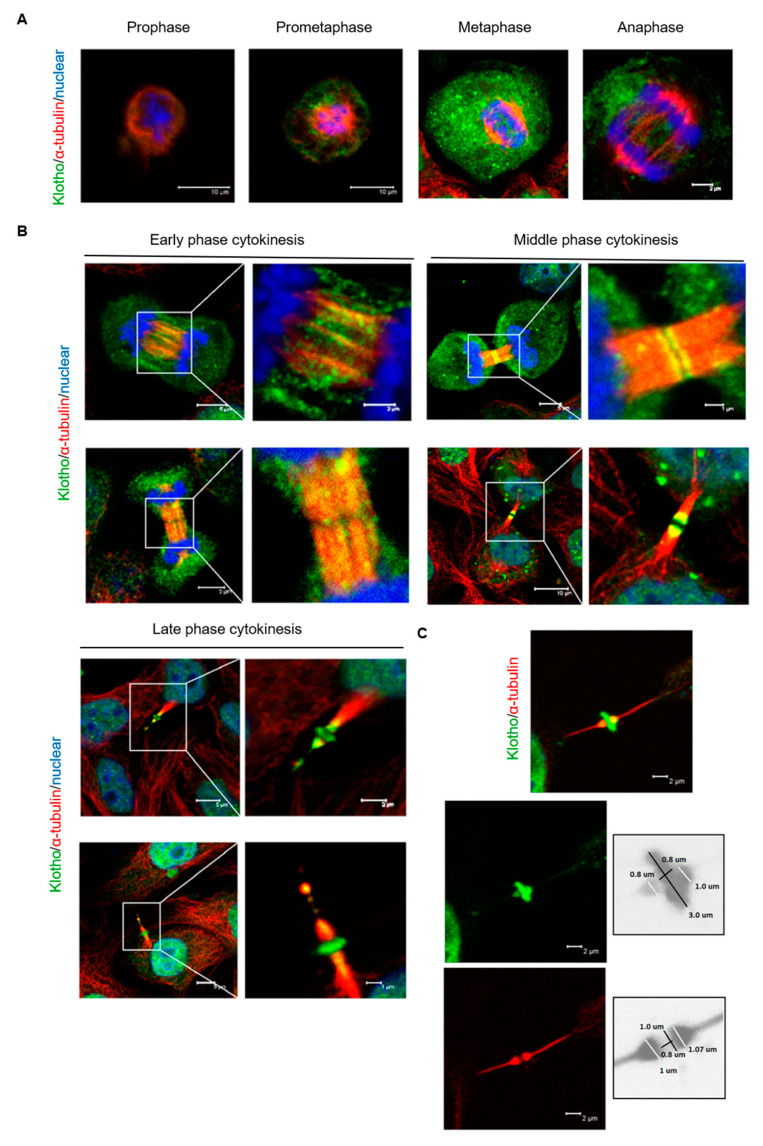
Cellular localization of endogenous Klotho protein during mitosis. Endogenous Klotho protein of cultured HeLa cells was analyzed by immunofluorescence with an N-terminal specific antibody for Klotho. (**A**) Cellular localization of Klotho during the mitotic process from the prophase to the anaphase. (**B**) Cellular localization of Klotho during the cytokinesis. (**C**) Highlight of Klotho ring in the cytokinesis midbody (microscopy: ×400).

To determine the subcellular localization of Klotho in mitotic cells (HeLa cells), endogenous Klotho were analyzed by immunofluorescent staining. During the mitosis, the expression of Klotho was unevenly distributed as a dot-like structure in the cytoplasm; meanwhile, membrane localized Klotho was noted (Figure 3A). During the early cytokinesis stage, Klotho was found to be condensed on the zones of the cytokinesis bridge (Figure 3B). Klotho was also found to be localized at the midbody during middle and late stage cytokinesis (Figure 3B). A cone-like structure at the outer edges of the midbody and a ring structure at the midbody zone of Klotho were noted during late cytokinesis. The estimated diameters of the Klotho cone and ring were 1.0 and 3.0 μm, respectively (Figure 3C). These results suggest that Klotho was associated with cytokinesis progression. 

**Figure 4:**
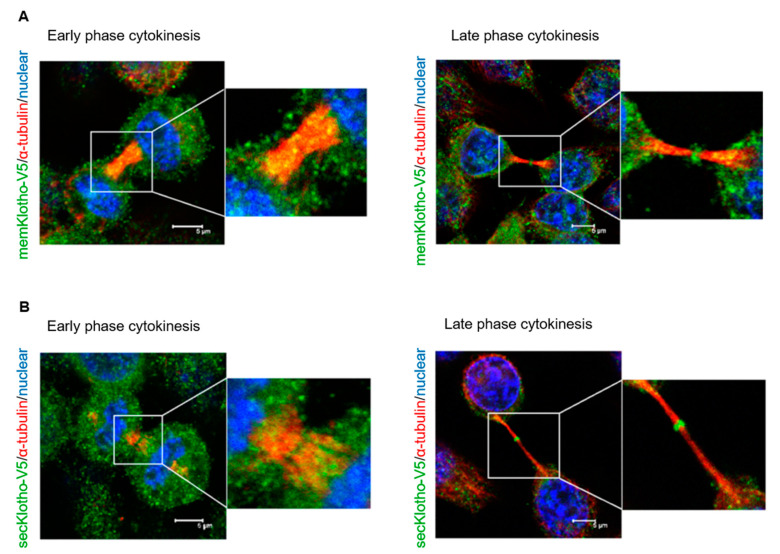
Cellular localization of secreted and membrane Klotho proteins during cytokinesis. Cultured HeLa cells transfected with memKL-V5 (**A**) or SecKL-V5 (**B**) expression plasmids for 24 h were analyzed by immunofluorescence with an anti-V5 antibody. The representative images of early and late cytokinesis are shown (memKL-V5: membrane Klotho; SecKL-V5: secreted Klotho; microscopy: ×400).

It is known that two Klotho isoforms, membrane and secreted forms, are formed by RNA alternative splicing or protease cleavage [21]. For defining the subcellular localization of the membrane and secreted forms of Klotho during cytokinesis, immunofluorescent staining with HeLa cells with the membrane Klotho or secreted Klotho overexpression was done. The results showed that the membrane (Figure 4A) and secreted Klotho (Figure 4B) had a similar subcellular localization during the cytokinesis process.

### 3.3. Klotho Depletion Induced Cytokinesis Bridge Abscission Delay

**Figure 5:**
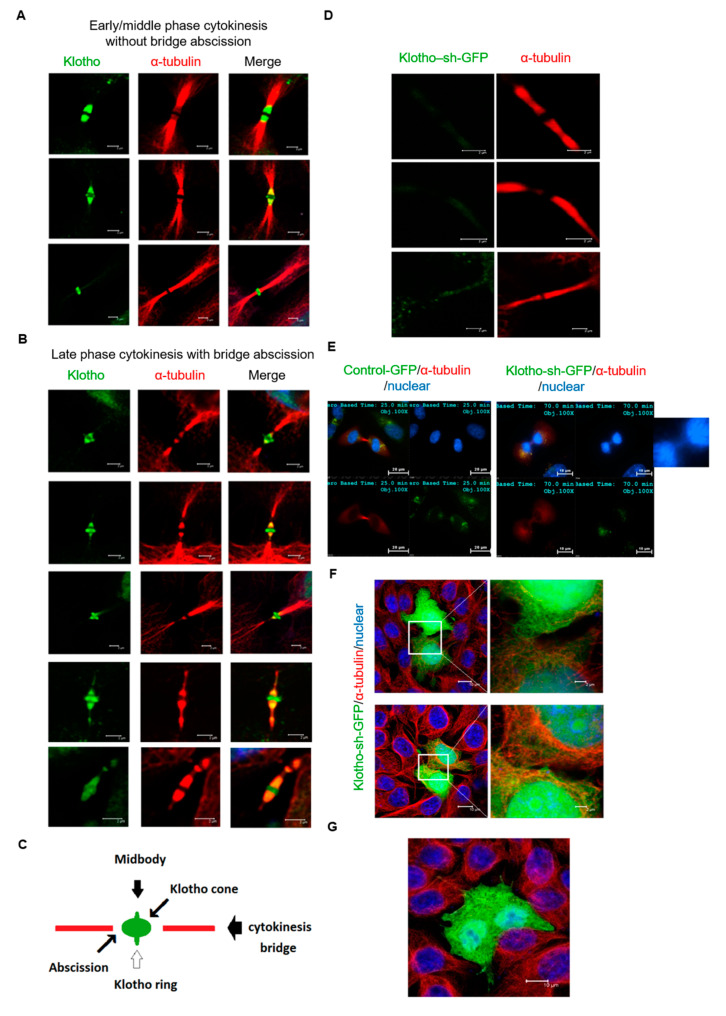
Klotho depletion impairs cytokinesis bridge abscission. The intrinsic Klotho of cultured HeLa cells was analyzed by immunofluorescence with Klotho N-terminal specific antibody for (**A**,**B**). (**A**) Cytokinesis bridge structures of early and middle stages of cytokinesis. (**B**) Cytokinesis bridge structures of late stages of cytokinesis. (**C**) Illustration of the space association between the Klotho ring and cone, and cytokinesis bridge abscission. Cultured HeLa cells transfected with control-GFP or Klotho-sh-GFP were analyzed by immunofluorescence for (**D**–**G**). (**D**) Tubulin immunofluorescence staining with HeLa cells transfected with Klotho-sh-GFP. The representative images of the bridge of late cytokinesis are shown. (**E**) Live cell images of HeLa cells at late cytokinesis stage. The white arrow box indicates the delayed chromatin separation in Klotho depleted cells. (**F**) Representative image of Klotho depleted HeLa cells with connected cytoplasm. (**G**) Representative image of Klotho depleted HeLa cells with multiple nuclei. The ratios of multinucleated cells of wild type and Klotho depleted HeLa cells under the normal growth condition are calculated. The ratios of multinucleated cells are 5% (1/20) and 16.7% (3/18) for wild type and Klotho depleted cells, respectively (microscopy: ×400).

To further investigate the association of cytokinetic abscission and Klotho localization, the spatial correlation between the abscission site and the localization of Klotho was analyzed by immunofluorescent staining. The results indicated that Klotho was localized at the body and outer edges of the midbody in the early to middle stage cytokinesis before bridge abscission starting (Figure 5A). At the late cytokinesis stage, Klotho was observed to localize at both ends of the midbody and next to the abscission sites. The immunofluorescence of the abscission midbodies showed that the cortical contraction of abscission occurred near the border of high Klotho intensity regions (Figure 5B). The spatial correlation between the abscission site and the localization of Klotho is summarized in Figure 5C. 

To explore the roles for Klotho, we monitored the cytokinesis abscission with HeLa cells with Klotho depletion. The study results indicated that the cytokinesis bridge structure is unusually elongated between divided cells. In addition, the abscission sites were not observed in the Klotho depleted cells in the late stage of cytokinesis (Figure 5D). The live cell analysis show that the mitosis process of the control cells lasted for about 90 min. In contrast, the cytokinesis of the Klotho knockdown cells was prolonged for 6 h. The live cell study also demonstrated that cells with Klotho depletion had significantly prolonged cytokinesis (Supplemental Movie 1: control-GFP; Supplemental Movie 2: Klotho-sh-GFP). It was also found that impaired cytokinesis caused by Klotho depletion might result in the incomplete separation of nuclear (Figure 5E) and cytoplasmic contents (Figure 5F). In addition, multinuclear was observed in the Klotho depleted cells (Figure 5G).

### 3.4. Klotho Depletion Impairs Aurora Kinase Activation

**Figure 6:**
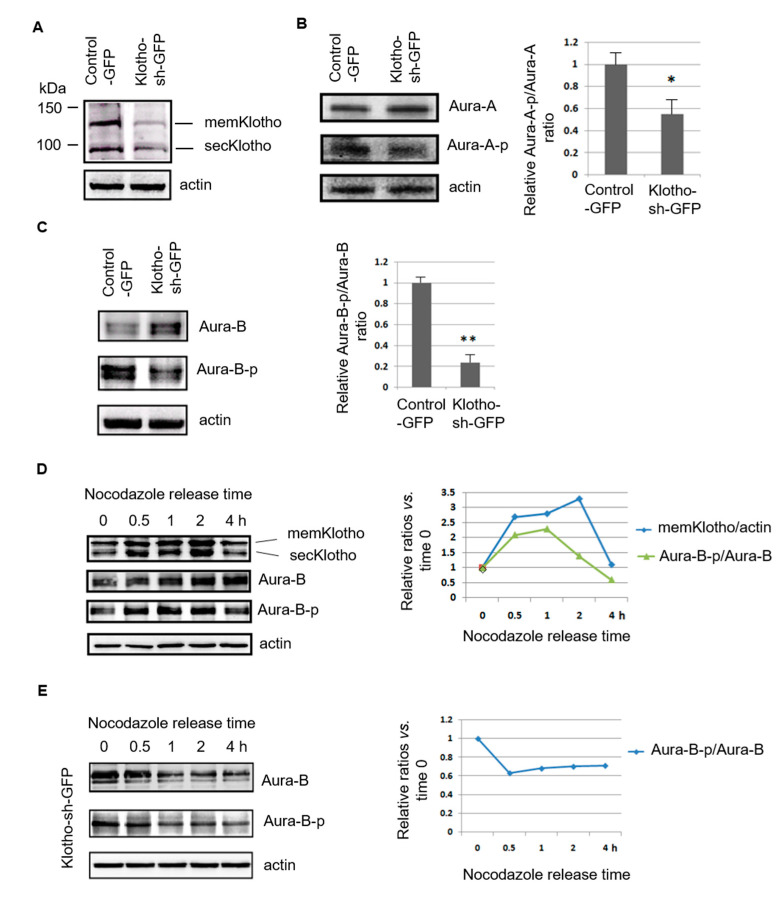
Klotho depletion impairs Aurora kinase activation. (**A**–**C**) Immunoblot analysis of Klotho, and Aurora kinase A and B with the protein extracts of transfected HeLa cells, respectively. (**D**,**E**) Immunoblot analysis of Klotho and Aurora kinase B with the protein extracts of HeLa cells treated with nocodazole for 16 h, respectively. Each treatment for (**B**,**C**) was repeated in triplicate, and the statistic results are plotted (means ± SEM; one-way ANOVA; * *p* < 0.05; ** *p* < 0.01; KL—Klotho; Aura A—Aurora kinase A; Aura B—Aurora kinase B).

The reactome pathway analysis suggested that Klotho might have roles in Aurora kinase A activation. In addition, a potential physical interaction between Klotho and Aurora kinase B (protein ID: C7G535) was noted by the IP/LC/MS/MS study (Supplementary information, Table S1a). Aurora kinase A and B are members of a family of mitotic serine/threonine kinase, and are implicated with important processes during mitosis [22]. The Western blotting results indicated that Klotho knock down by transfection with Klotho-sh-plasmid could inhibit the phosphorylation of Aurora kinase A and B in regularly grown HeLa cells (Figure 6A–C). The Klotho expression increased soon after the cells were released from the nocodazole synchronization and they were correlated with Aurora kinase B activation (Figure 6D). In contrast, the phosphorylation of Aurora kinase B decreased soon after releasing from the nocodazole synchronization in cells with Klotho depletion (Figure 6E).

### 3.5. The Spatial Localization of Klotho in the Midbody Relating with Cytokinetic Protein Kinases

**Figure 7:**
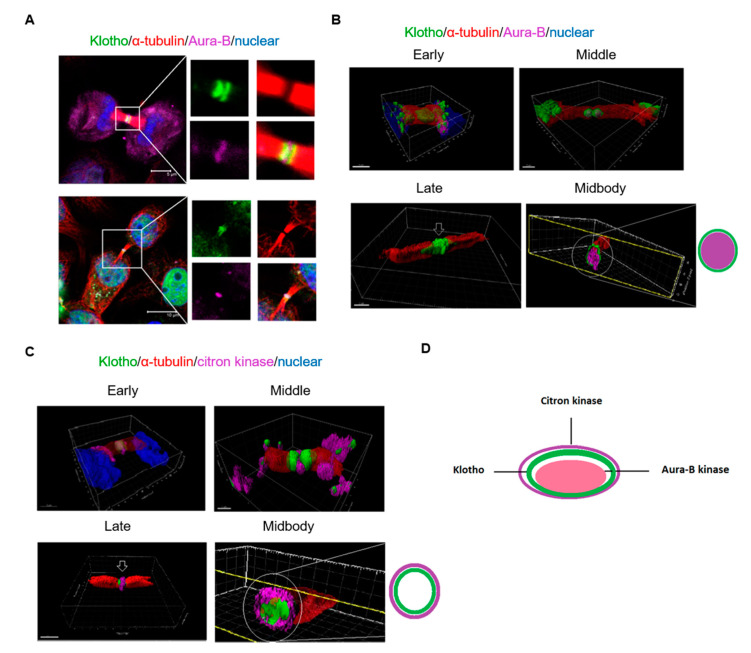
3D reconstruction of the cytokinesis bridge. Intrinsic Klotho, Aurora kinase B, and citron kinase of cultured HeLa cells analyzed by immunofluorescence. (**A**) Results of immunofluorescence staining for Klotho and Aurora kinase B. (**B**) 3D structure of Klotho and Aurora kinase B in the cytokinesis bridge. (**C**) 3D structure of Klotho and citron kinase in the cytokinesis bridge. (**D**) Midbody architecture related with Klotho, Aurora kinase B, and citron kinase (microscopy: ×400).

For detailing the spatial localization of Klotho in the midbody and its correlation with cytokinesis related protein kinase, immunofluorescent staining for intrinsic Klotho and three dimensional (3D) reconstruction were performed. The results demonstrated that the midbody localization of Aurora kinase B happened in the early to middle stage of cytokinesis (upper panel of Figure 7A). At the late cytokinesis stage, Klotho colocalized with the Aurora kinase B in the midbody (bottom panel of Figure 7A). The 3D reconstruction results indicated that the Aurora kinase B was covered by the Klotho ring in the midbody (Figure 7B; Supplemental Movies 3a–c). We also performed the immunofluorescent staining for another cytokinesis related kinase, citron kinase. Citron kinase has important roles in regulating midbody formation and cytokinesis bridge abscission [23,24]. A cross-regulation between Aurora kinase B and citron kinases has been reported to be crucial in controlling the midbody architecture in cytokinesis [25]. The 3D reconstruction results indicated that Klotho was covered by the citron kinase in the midbody at the late stage of cytokinesis. It was also found that midbody localization of citron kinase happened earlier than Klotho (Figure 7C; Supplemental Movies 4a–c). The spatial correlations of Klotho related with Aurora kinase B and citron kinases in the midbody are summarized in Figure 7D. Based on these observations, we suggested that Klotho plays important roles in cytokinesis regulation. We performed immunofluorescent staining for Aurora kinase B with Klotho depleted HeLa cells. The staining results showed Klotho depletion did not significantly impair the Aurora kinase B’s localization during cytokinesis (Supplemental information, Figure S1). It is suggested that Klotho might regulate kinase activity rather than localization during cytokinesis.

### 3.6. Klotho Depletion Causes Embryo Cleavage Defects in Zebrafish

**Figure 8:**
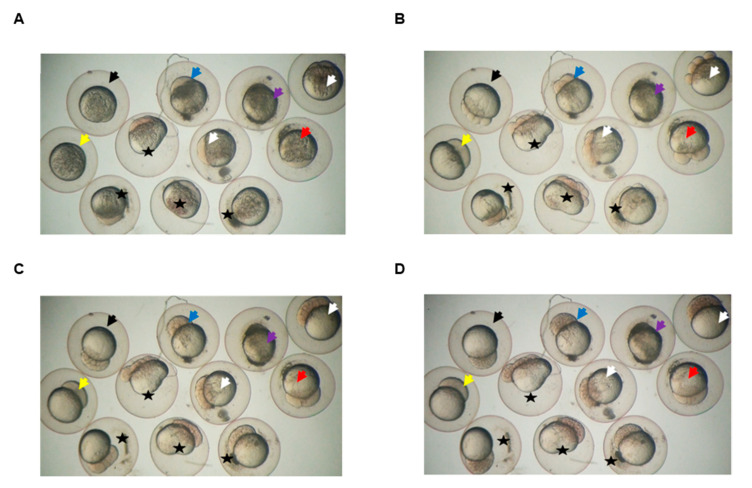
Klotho knockdown causes cleavage defects in zebrafish embryos. Images were captured from live video (Supplemental Movie 5) of wild type zebrafish embryos without any treatment (uninjected, black arrow) or after injection with dye (mock, blue arrow), or dye plus Klotho-morpholino. Most of the Klotho-morpholino-injected embryos displayed defective phenotypes, including no cleavage (yellow arrow), cleavage stopped at two cells (red arrow), and asymmetrical cleavages (stars). Some embryos died (purple arrow), and others developed normally (white arrow). (**A**): One to two cells stage; (**B**): four to eight cells stage; (**C**): 32 to 64 cells stage; and (**D**): around 128 cells stage.

Cytokinesis is a critical process for controlling embryo cleavage and establishing embryonic cell polarity [26,27]. For defining the potential effects of Klotho on cytokinesis in vivo, the fertilized zebrafish eggs with Klotho-MO injection were used for the study. Western blotting analysis showed that endogenous zebrafish Klotho was strongly detected in both the uninjected- and control-MO-injected-groups. In contrast, Klotho signals appeared in a weaker manner in the Klotho-MO-injected group (Supplemental information, Figure S1). These results suggested that endogenous zebrafish Klotho protein expression was down regulated by Klotho-MO. Furthermore, our results also showed that abnormal cell cleavage at the early developmental stages were observed (~86.7%; 52/60; Figure 8). The abnormal cell cleavage phenotypes included no cleavage, cleavage stopped at two cells, and asymmetrical cleavages. The live video for the early embryonic development of uninjected, mock, control-MO-, and Klotho-MO-injected groups are added as supplemental movies (Supplemental Movie 5 and 6). Taken together, we proposed that knockdown of Klotho impaired cell cleavages in zebrafish embryos.

## 4. Discussion

**Figure 9:**
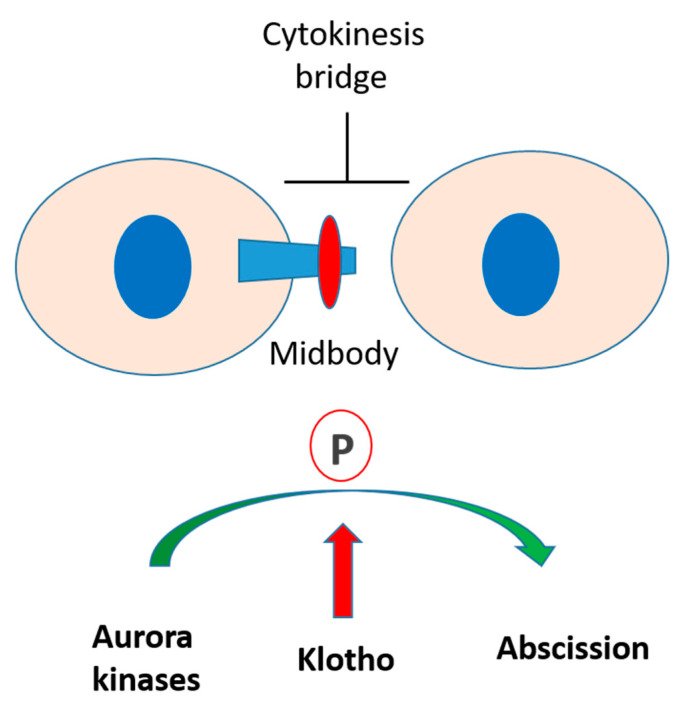
Putative roles of Klotho in cytokinesis. Klotho is essential for cytokinesis by regulating Aurora kinase activation. Klotho depletion inhibits Aurora kinase activation and results in impaired cytokinesis bridge abscission.

Recent evidence indicates that Klotho has important roles in cell proliferation and differentiation [9,28,29]. In vitro and in vivo studies have reported that FGF23/Klotho signaling could stimulate cell proliferation by activating MAPK, Cyclin D1, and c-myc pathways [9,28]. However, little is known concerning its potential role on the mitosis process. We reported here that Klotho is essential for cytokinesis by regulating Aurora kinase activation (Figure 9). Klotho depletion resulted in an aberrant cell cycle and delayed cytokinesis. The in vivo study with fertilized zebrafish eggs also indicated that Klotho knockdown caused disoriented cell division in the early stage of embryo development. These results indicate that Klotho plays an important role in cytokinesis process. 

Our study further demonstrated that Klotho is co-localized with Aurora kinase B and citron kinases in the midbody during the cytokinesis, and is correlated with cytokinesis bridge abscission. The midbody is composed of several proteins localized to the preceding contractile ring and central spindle, which show a very precise and stereotypic distribution [30,31]. The midbody is important for completing the final stages of cytokinesis, abscission [32]. Aurora kinases have been implicated in several vital events in mitosis. Accumulated evidence indicates that Aurora kinases are master regulators of late mitotic events and cytokinesis. Aurora kinase A is mainly associated with midbody during telophase. As cells progress from anaphase to the completion of cytokinesis, Aurora kinase B re-localizes to the spindle midzone and midbody [33]. Deregulation of Aurora kinase activity can result in mitotic abnormality and genetic instability, leading to defects in centrosome function, spindle assembly, chromosome alignment, and cytokinesis. Aurora kinase B and citron kinases are determinant kinases for cytokinesis [22]. The Aurora kinase B, which is essential for cytokinesis, stabilizes and bundles overlapping anti-parallel microtubules in the midzone [34,35]. A spatial phosphorylation gradient of Aurora kinase B in the midzone provides spatial information for events in anaphase and cytokinesis [36]. It has been reported that Aurora kinase inhibition causes a disruption of midzone organization [37]. Citron kinase, a target of GTPase Rho, is considered to contribute to cytokinesis. Mutated citron causes abnormal contractions during cytokinesis, and results in the formation of multinucleated cells [38]. Previous studies have demonstrated that cross-regulation between Aurora B and citron kinase is important for controlling midbody architecture in cytokinesis. Citron kinase could promote Aurora kinase B activity through phosphorylation of the INCENP CPC subunit. In turn, Aurora kinase B controls citron kinase localization through phosphorylation during cytokinesis [25]. In addition, a recent study demonstrated that Aurora kinase B activity is required for the downstream activation of RhoA, which is essential for cytokinesis abscission [39]. The present study shows that Klotho had a specific stereotypic distribution to separate Aurora kinase B and citron kinase in the midbody. We also found that Klotho depletion impairs Aurora kinase activation. These results suggest that the Klotho might have determinant roles in the cross-regulation between kinases in cytokinesis. 

Cytokinesis is the final stage of cell division. Cytokinesis failure in mammals is associated with carcinogenesis. Cell cleavage failure caused by impaired cytokinesis can lead to genetic instability and potentially to tumorigenesis [40]. Accumulated evidence indicates that Klotho acts as tumor suppressor in many cancer cell types, such as pancreas cancer, colon cancer, and hepatoma. Klotho overexpression could inhibit tumor cell proliferation and induce cell cycle arrest [41,42,43]. Our study revealed that Klotho depletion resulted in abnormal cytokinesis. It is suggested that maintaining normal cell cycle and division might be an important tumor suppression mechanism of Klotho.

## 5. Conclusions

Our observation indicated that Klotho might have a critical role in cytokinesis regulation by interacting with the cytokinesis related kinases. Much remains to be done to characterize the roles of Klotho in cytokinesis. Of particular interest is the question of how Klotho is recruited during cytokinesis and regulates the abscission machinery.

## Supplementary Materials

Supplementary materials can be found at https://www.mdpi.com/2073-4425/11/9/1048/s1. Movie 1: Cytokinesis live video of HeLa cells transfected with the control-GFP (control-GFP: green; alfa-tubulin: red; nuclear: blue). Movie 2: Cytokinesis live video of HeLa cells transfected with Klotho-sh-GFP (Klotho-sh-GFP: green; alfa-tubulin: red; nuclear: blue). Movie 3: 3D structure of Klotho and Aurora kinase B in the bridge. (a) Early cytokinesis; (b) middle cytokinesis; (c) late cytokinesis (Klotho: green; Aurora kinase B: purple; alfa-tubulin: red; nuclear: blue). Movie 4: 3D structure of Klotho and citron kinase in the bridge. (a) Early cytokinesis; (b) middle cytokinesis; (c) late cytokinesis (Klotho: green; citron kinase: purple; alfa-tubulin: red; nuclear: blue). Movie 5: Early embryonic development in normal and Klotho knockdown zebrafish. Movie 6: Early embryonic development in uninjected, control-MO-injected, and Klotho-MO-injected zebrafish. Supplemental Table S1: Results of IP/LC/MS/MS and pathway analysis. (a) List of protein targets identified by IP/LC/MS/MS analysis (two peptide identified, > two-fold increase, Klotho Ab vs. non-specific IgG). (b) List of reactome pathways with *p* value less than 0.05. Supplemental Figure S1: (A) Western blotting analysis of Klotho protein expression levels in the zebrafish embryos without any treatment by injection with different morpholinos (CTL: control morpholino, random sequence; Klotho-MO: anti-sense to zebrafish *klotho* gene translation start site). (B) All of the pictures were captured from Movie 6. The results showed that the embryos divided normally in the uninjected and CTL-MO-injected group, but impaired cell division in the Klotho-MO-injected group (arrow indicates).

## Abbreviations

KL	Klotho
hpf	Hours post-fertilization
GFP	Green fluorescent protein
MO	Morpholino
